# Building a Biopsychosocial Conceptual Framework to Explore Pressure Ulcer Pain for Hospitalized Patients

**DOI:** 10.3390/healthcare4010007

**Published:** 2016-01-08

**Authors:** Junglyun Kim, Hyochol Ahn, Debra E. Lyon, Joyce Stechmiller

**Affiliations:** Department of Family, Community and Health System Science, University of Florida College of Nursing, P.O. BOX 100197, Gainesville, FL 32610-0197, USA; genajustin@ufl.edu (J.K.); delyon@ufl.edu (D.E.L.); stechjk@ufl.edu (J.S.)

**Keywords:** biopsychosocial model, pressure ulcer pain, conceptual framework, hospitalized patients

## Abstract

Although pressure ulcers are a prevalent condition, pain associated with pressure ulcers is not fully understood. Indeed, previous studies do not shed light on the association between pressure ulcer stages and the experience of pain. Especially, pain characteristics of suspected deep tissue injury, which is a new category that was recently added by the National Pressure Ulcer Advisory Panel, are yet unknown. This is concerning because the incidence of pressure ulcers in hospitalized patients has increased exponentially over the last two decades, and health care providers are struggling to ensure providing adequate care. Thus, in order to facilitate the development of effective interventions, this paper presents a conceptual framework to explore pressure ulcer pain in hospitalized patients. The concepts were derived from a biopsychosocial model of pain, and the relationships among each concept were identified through a literature review. Major propositions are presented based on the proposed conceptual framework, which integrates previous research on pressure ulcer pain, to ultimately improve understanding of pain in hospitalized patients with pressure ulcers.

## 1. Introduction

Pressure ulcers are a significant and highly prevalent health care problem in the United States, especially among hospitalized patients. The number of hospitalizations related to pressure ulcers has increased by 86.4% between 1993 and 2008. Moreover, pressure ulcers affected 503,300 hospitalized patients and cost more than $11 billion in 2006 alone [1]. The incidence of hospital acquired pressure ulcers was 4.5% between 2006 and 2007 [2], and the costs of hospitalization for Stage III and IV pressure ulcers was $43,180 per case [3].

In response to the incidence and rising costs, in 2008 the Centers for Medicare and Medicaid Services (CMS) identified advanced pressure ulcers acquired during hospitalization as preventable conditions, rendering hospitals unable to receive Medicare payments or private insurance reimbursement for the care of patients with advanced stages of pressure ulcers [4]. As a result of this policy, the Agency for Healthcare Research and Quality estimated a 23% decrease in hospital acquired pressure ulcers from 2011 to 2014, which led to a savings of approximately $5,270,000,000 as well as a 22,444 death reduction in hospitals [5]. Despite decreasing incidence and costs of hospital acquired pressure ulcers, this improvement reflects only a short term period.

According to statistics from the National Center for Health Statistics (NCHS) posted by the Center for Disease Control and Prevention (CDC), 159,000 nursing home residents in the United States have pressure ulcers, which is approximately 11% of the total nursing home population [6]. Many of these pressure ulcers go untreated. In fact, in patients with a Stage II or higher pressure ulcer, only 34% of the population receive special wound care [6]. This is concerning because several qualitative studies have shown that pressure ulcers and their treatments adversely affect patients. For example, Gorecki and colleagues (2009) reported that pressure ulcers may result in constant physical pain, reduced social activity, and increased care costs [7]. Similarly, Hopkins and colleagues [8] as well as others found that pressure ulcers often cause unremitting pain and severe restrictions of the activities of daily living [9]. Patients often describe wound pain as “constant” and “burning hotly” [10] and wound care causes pain [11]. Consequently, healthcare providers need to respond to pain with pressure ulcers quickly [12], and provide adequate treatment of pain during wound care [13] to ensure they are providing adequate care.

The National Pressure Ulcer Advisory Panel defines pressure ulcers as localized injuries to the skin and/or underlying tissue, usually over a bony prominence, as a result of pressure or pressure in combination with shear [14]. Pressure ulcers form when prolonged pressure damages tissue over areas of bony prominence due to decreased oxygen supply and nutrition delivery [15]. Coleman and colleagues [16] reported that the risk factors for pressure ulcers include immobility, general skin status, poor perfusion, poor sensory perception/response, diabetes, poor nutrition, moisture, and low albumin. According to the National Pressure Ulcer Advisory Panel (NPUAP) [14], pressure ulcers have four stages and two additional categories: Stage I is characterized by non blanchable, localized erythema; Stage II is characterized by shallow open ulcers without slough; Stage III is characterized by full thickness skin loss; Stage IV is characterized by full thickness tissue loss; “unstageable” is characterized by full thickness tissue loss in which the actual depth of the ulcer is unknown; and suspected deep tissue injury (sDTI) is characterized by purple or maroon discoloration with intact skin. Suspected DTI, which was recently added as a pressure ulcer category by the NPUAP, is caused by bony compression, deformation, and distortion of the deep tissue, and results in severe ischemia and painful compression injuries [16].

Although pressure ulcers are highly prevalent in hospitalized patients [1,6], the relationship between pain and pressure ulcer stages/categories is not fully understood. To address this gap in knowledge, in 2008 Gunes [10] outlined the characteristics of pain in pressure ulcers: hot burning (across all stages), throbbing (Stage II), tender and stabbing (Stage III), and throbbing, heavy, and sharp (Stage IV). Furthermore, pain at all stages was described as tender and burning; Stage II and III pain was described as sore; Stage II and IV as stinging; and Stages III and IV as stabbing [17]. According to the literature review by Girouard and colleagues [18], few studies have examined the relationship between pressure ulcer stages/categories and pain. In addition, results have revealed conflicting findings about pain associated with each stage of pressure ulcers. For example, one study indicated that advanced pressure ulcers are less painful than lower staged pressure ulcers [18], whereas other studies indicated that advanced pressure ulcers are more painful than lower staged pressure ulcers [9,10,19,20]. In response to these findings, McGinnis and colleagues [21] suggest that pain is not relevant to pressure ulcer stages. This view is reflected in current guidelines for pressure ulcer pain, which suggest that health care providers simply assess pain associated with pressure ulcers, minimize friction to encourage position change, and provide adequate pain control during dressing change [22,23]. Clearly, existing studies do not fully explain the pain experiences of patients with pressure ulcers, and no guiding conceptual framework exists in the literature that fully describes the pain experiences of this population. Thus, to improve the understanding of pain experiences related to pressure ulcer stages/categories, we aimed to develop a conceptual framework to guide research and practice, as well as develop pain management plans for hospitalized patients with pressure ulcers.

This paper proposes a biopsychosocial conceptual framework to examine the relationship between pain and pressure ulcers, and shows empirical evidence for the modifiers of pain experiences. More specifically, this paper aims to: (1) explore relationships between pressure ulcers stages/categories and pain experiences in hospitalized patients; (2) explain influences of biological, psychological, sociocultural, and environmental factors of the pain experiences related to pressure ulcers in hospitalized patients. This framework will advance research about pain associated with pressure ulcers and provide a conceptual framework to promote the development of therapeutic interventions to decrease pain in populations with pressure ulcers.

## 2. Method

In order to develop a conceptual framework to explain pain experiences in patients with pressure ulcers, we first identified relevant concepts and the relationships among the concepts through an extensive literature review. Next, we conducted an extensive literature search for theories with relevant concepts and selected a parent model. Finally, we established relationships between concepts and the parent theory, and modified the parent model to build the theoretical framework.

Concepts were identified from the literature review that explain pain phenomena. Literature was searched in CINAHL and PubMed using the key word “pain” along with the words “physical”, “psychological”, “cultural”, and “pressure ulcers”. The inclusion criteria were peer reviewed articles published before 2015 in English. The identified concepts were labeled as meanings under related constructs. The methods supported the aim of the conceptual framework: (1) to describe pain experiences related to pressure ulcers; (2) to explain the predictors and moderators of pain experiences based on existing evidence; and (3) to predict pain characteristics in patients with different stages/categories of pressure ulcers [24]. Several conceptual models which were able to explain pressure ulcer pain were found: the gate theory [25], the nociceptive theory [26], the pain assessment model [27], the lens model [28], and the biopsychosocial model of pain [29]. After reviewing related literature, the biopsychosocial model of pain [29] was chosen as the parent conceptual model. The parent model was selected based on meaning, logical adequacy, usefulness, generality, parsimony, and testability [24].

**Figure 1:**
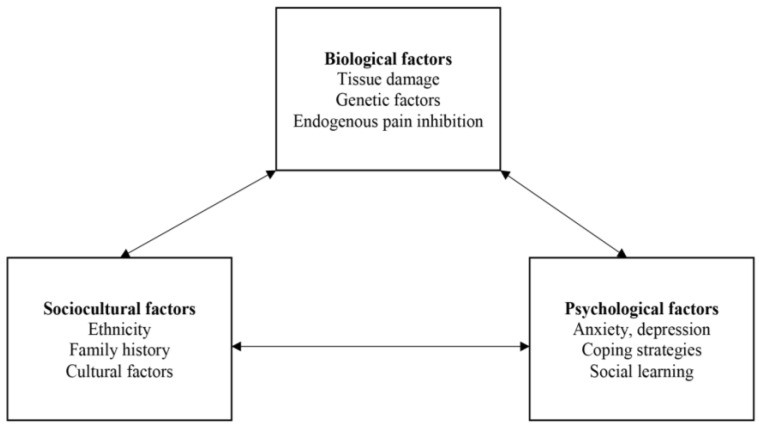
The Biopsychosocial Model of Pain [29].

The biopsychosocial model of pain was chosen because it contains the most relevant concepts to explain pressure ulcer pain. Indeed, it depicts pain as a unique, individual experience, and a consequence of complex, biopsychosocial interactions. The three main constructs of the biopsychosocial model of pain are: (1) biological factors such as tissue damage, genetic factors, and endogenous pain inhibition; (2) sociocultural factors such as ethnicity, family history, and cultural factors; and (3) psychological factors such as anxiety, depression, coping strategies, and social learning (Figure 1). The diagram explains individual variability in pain perception and experiences as a consequence of interactions among the variables. After selecting the parent model, we revised the biopsychosocial model of pain by adding environmental factors (e.g., nurse/patient ratio, nurse educational level, frequency/type of wound dressing) and modifying psychological factors (*i.e.*, we added anger, stress, sleep, fatigue and catastrophizing, and withdrew social learning), biological factors (*i.e.*, we added comorbidity, inflammation, infection, and age, and withdrew tissue damage) and social cultural factors (*i.e.*, we added discrimination and social support, and withdrew family history and cultural factors).

## 3. Results

Concepts of pain experiences and the relationships between concepts were derived from the biopsychosocial model of pain. The key concepts of the proposed conceptual framework are pressure ulcer stages/categories as inputs and pain experiences as an outcome. Other concepts are sociocultural, psychological, and biological factors, which may moderate the pain associated with pressure ulcers. Similarly, environmental factors have been added because the target population is hospitalized patients.

**Figure 2:**
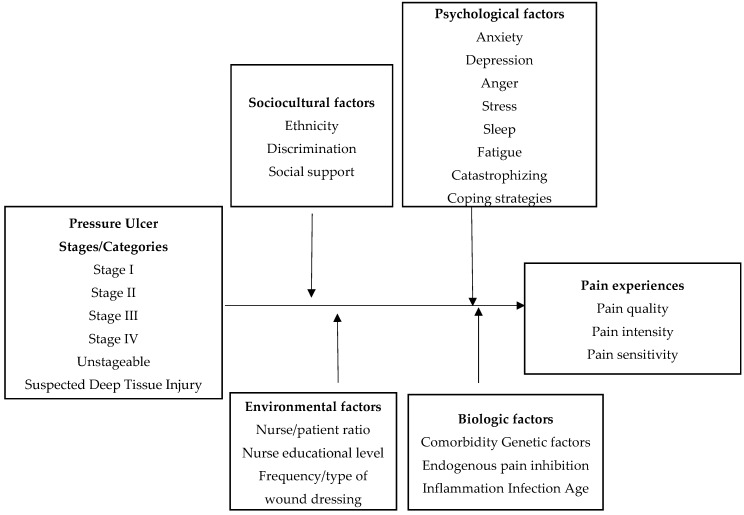
The Proposed Conceptual Framework.

The proposed conceptual framework contains the following concepts (Figure 2): (1) pressure ulcer stages/categories as inputs; (2) pain experiences as an outcome; (3) comorbidities, genetic factors, endogenous pain inhibition, inflammation, infection, and age as biological factors; (4) ethnicity, discrimination, and social support as sociocultural factors; (5) anxiety, depression, anger, stress, sleep, fatigue, catastrophizing, and coping strategies as psychological factors; and (6) nurse/patient ratio, nurse educational level, and frequency/type of wound dressing as environmental factors. The definitions of these concepts are summarized in Table 1. The detailed discussion of the empirical indicators is beyond the scope of this paper. This paper is focused on the presentation and relationships among concepts.

**Table 1:** Factors and components of the proposed model.

Factors and Components	Definitions	Possible Empirical Indicators
**Pressure Ulcer**		
Pressure ulcer stages/categories	Stage of localized injury to the skin and/or underlying tissue, usually over bony prominence, as a result of pressure, or pressure in combination with shear	National Pressure Ulcer Advisory Panel (NPUAP) Pressure Ulcer Stages/categories
**Biological Factors**		
Comorbidity	Two or more coexisting medical conditions or unrelated disease processes	Charlson Comorbidity Index (CCI)
Genetic factors	The factors pertaining to or produced by a gene	catechol-O-methyltransferase gene (COMT), and mu-opioid receptor gene (OPRM1)
Endogenous pain inhibition	The pain inhibition originating from within the body	Quantitative Sensory Testing
Inflammation	The protective response of body tissue to irritation or injury	C-reactive protein, tumor necrosis factor-alpha, and interleukins
Infection	The invasion of the body by pathogenic microorganisms that reproduce and multiply, causing disease by local cellular injury, secretion of a toxin, or antigen-antibody reaction in the host	Plantonic bacteria and biofilm
Age	The number of years of life	Years of life
**Sociocultural Factors**		
Ethnicity	Discrete groups of people that are similar according to behaviors, culture, and biophysical characteristics	Multigroup Ethnic Identity Measure (MEIM)
Discrimination	Unfair or different treatment based on membership in a group	Experience of Discrimination Scale (EOD)
Social support	The availability of resources from others who are connected by social networks to an individual	Multidimensional Scale of Perceived Social Support
**Psychological Factors**		
Anxiety	Anticipation of impending danger and dread accompanied by restlessness, tension, tachycardia, and breathing difficulty not associated with an apparent stimulus	The State and Trait Anxiety Inventory (STAI)
Depression	A condition of loss of interest or pleasure in life activities that cause significant impairment in social, work, or other important areas of functioning	Patient Health Questionnaire (PHQ9) and Center for Epidemiological Studies-Depression Scale (CES-D)
Anger	An emotional reaction characterized by extreme displeasure, rage, indignation or hostility	The Anger Regulation and Expression Scale (ARES), and State Trait Anger Expression Inventory (STAXI)
Stress	Stressors or anxiety that acts as a stimulus or events that evoke physiologic responses	Perceived Stress Scale (PSS)
Sleep	A state marked by reduced consciousness, diminished activity of skeletal muscles and depressed metabolism	Pittsburg Sleep Quality Index (PSQI), Sleep Hygiene Practice Scale (SHPS), and Insomnia Severity Index (ISI)
Fatigue	A nonspecific, common subjective feeling of low vitality that disrupts daily functioning	Fatigue Severity Scale (FSS)
Catastrophizing	A negative cognitive and affective process involving magnification of pain-related symptoms, helplessness, pessimism, and rumination about pain	Pain Catastrophizing Scale (PCS)
Coping strategy	The cognitive and behavioral adjustments that an individual uses to confront and manage health and daily-life stressors	Coping Strategies Questionnaire-Revised (CSQ-R)
**Environmental Factors**		
Nurse/patient ratio	The number of patients assigned to RN	Number of patients per RN
Nurse educational level	The level of education of the nurse	Educational Preparation
Frequency/type of wound dressing	The number of dressing changes/type during 24 h	Frequency/type of dressing per a day
**Pain Experiences**		
Pain intensity	The level of intensity of pain	Numerical Rating Scale (NRS), Visual Analog Scale (VAS), and Wong-Baker FACES Pain Rating Scale
Pain quality	Any of the features that make pain	McGill Pain Questionnaire
Pain sensitivity	A condition of being sensitive to pain	Heat pain threshold/tolerance, cold pain threshold/tolerance, and pressure pain threshold

### 3.1. Pain Quality, Intensity, and Sensitivity

Pain is an unpleasant sensory and emotional experience evoked by tissue damage [30]. It is an outcome variable in the proposed conceptual framework, which uses pain quality, intensity, and sensitivity to measure pain phenomena in patients with pressure ulcers. In 2011, Gorecki and colleagues [17] stated that descriptions of pressure ulcer pain vary according to pressure ulcer stages: “tender” and “burning” are used to describe all stages of pressure ulcers; “sore” is used to describe Stages II and III; “stinging” is used to describe Stages II and IV; and “stabbing” is used to describe Stages III and IV. Similarly, Girouard and colleagues [18] and others described pressure ulcer pain as a burning sensation, and stated that patients with pressure ulcers experience various levels of pain from pressure ulcers [9,10,19,20]. Experimental pain sensitivity using quantitative sensory testing (QST) is used often to study patients with pain; however, QST has not been frequently used in studies about pressure ulcer pain. QST may be a useful way to measure pain hypersensitivity caused by central sensitization in patients with pressure ulcers [19]. Thus, in order to describe individual pain experiences related to pressure ulcers, future studies are recommended to include detailed measurements of pain. This conceptual framework gives clear and comprehensive understanding of pain related to pressure ulcers. Thus it will facilitate research for measurement of pain in pressure ulcer population.

### 3.2. Sociocultural Factors

Sociocultural factors, such as ethnicity, discrimination, and social support, influence pain [31]. Ethnicity, which refers to discrete groups of people with similar behaviors, culture, and biophysical characteristics [32], is one of the sociocultural contextual factors that influence an individual’s health-related beliefs and behaviors [33]. Indeed, studies have shown that pain responses vary across ethnic groups and that racial and ethnic minorities have higher experimental pain sensitivity and clinical pain intensity compared to Caucasians [34,35,36].

Another sociocultural factor, discrimination, refers to unfair or different treatment based on membership in a group [37]. In the clinical setting, health care providers may discriminate against patients by prescribing treatments based on ethnic group stereotypes. For example, in their literature review, Garbez and Puntillo [38] reported that inappropriate pain management may be related to demographic factors such as age, gender, and ethnicity. Significantly, Black patients were 31% less likely to receive opioids compared to Caucasian patients (odds ratio (OR) = 0.69, *p* < 0.001) among 18 million ED visits during 2003 to 2006 [39]. In a similar study that included 153,891 patients in the emergency department, Pletcher and colleagues [40] reported that white patients are more likely to receive opioids than black, Hispanic, and Asian patients (adjusted odds ratio [OR] 0.66, 0.69, and 0.79 respectively).

The last sociocultural factor, social support, refers to the availability of resources from others who are connected by social networks to an individual [41]. Lee and colleagues [42] suggested that social support may buffer pain among the elderly who have chronic pain symptoms; this is because elderly patients with higher social support reported lower pain (regression coefficient (β) = −0.04, *p* < 0.05) in a study that included 299 participants in a retirement community in central Florida. Similarly, Takai and colleagues [43] reported social support is an effective long term strategy with pharmacotherapy and physical activity for the management of chronic pain. Finally, Ferreira and Sherman [44] described that social support affected pain (r = −0.28, *p* < 0.05) among 72 older adults with chronic pain [44].

Based on the above findings, sociocultural factors have significant influences on pain experiences. Health care providers should be aware of individual ethnicity and consider available social support and possible discrimination in healthcare when they manage pain in pressure ulcer population.

### 3.3. Biological Factors

Biological factors (e.g., comorbidity, genetic factors, endogenous pain inhibition, inflammation, infection, and age) influence pain. Comorbidity is defined as “two or more coexisting medical conditions or unrelated disease processes” [45]. Indeed, among a half million hospitalized patients with pressure ulcers in 2006, 90% of adults were hospitalized for treatment of other primary conditions, rather than treatment for pressure ulcers [1]. Onubogu [46] found that a higher number of comorbidities was significantly correlated with more severe bodily pain (*r* = 0.13, *p* = 0.001) in 1592 community-dwelling older adults with chronic pain. In a study that included 29,132 participants with chronic pain, Caporali and colleagues [47] reported that pain was significantly worse (*p* < 0.0001) when patients had two or more comorbidities. Comorbidity interferes with the pain experience in pressure ulcer population. It decreases pain perception or intensifies pain. It is difficult to differentiate the origin of pain in hospitalized patients. Therefore, healthcare providers should approach pain associated with pressure ulcers as a complex experience.

Currently, pain research involving genomics suggests that genes may affect pain a patient’s experience of pain [48]. Indeed, emerging evidence suggests that genetic factors are related to pain experiences. For example, the catechol-*O*-methyltransferase gene (COMT) and mu-opioid receptor gene (OPRM1) may be associated with pain-induced mu-opioid receptor binding [49], experimental pain sensitivity [50,51], and risk of developing chronic pain [52].

Individual differences in endogenous pain inhibition are assumed to provide a plausible mechanistic predictor of pain experiences. For example, Bruehl and colleagues [53] found that individual differences in endogenous opioid inhibition of chronic pain were associated with the magnitude of acute reductions in pain ratings after pain medication administration. Gene and endogenous pain inhibition is highly individualized and cannot be generalized. Gene and endogenous pain inhibition support importance of customized pain management. Healthcare providers should always apply specific patients’ needs when managing pain in the pressure ulcer population.

Another biological factor, inflammation, may influence pain [54]. Indeed, several studies reported that inflammation mediators (e.g., cytokines) are closely involved with and regulate the pain process. Inflammation is defined as “the protective response of body tissue to irritation or injury” [45]. For example, in a study that included 32 participants, Starkweather and colleagues [55] reported that women with chronic pain had a higher level of C-Reactive Protein (*p* < 0.01), interleukin-13 (*p* < 0.02), and interleukin-7 (*p* < 0.02) than women having no pain. Similarly, the literature review by DeVon and colleagues [56] reported that higher levels of proinflammatory markers (e.g., C-reactive protein, tumor necrosis factor-alpha, interleukins) were associated with greater pain.

Infection is defined as the invasion of the body by pathogenic microorganisms that reproduce and multiply, causing disease by local cellular injury, secretion of a toxin, or antigen-antibody reaction in the host [45]. Infection occurs when microorganisms invade the host tissue, causing injury to cells and host immunological reactions that defeat the host’s defensive mechanisms [57]. In a study with 44 participants with post burn, Juozapaviciene and colleagues [58] reported that patients with wound infections in the acute hospitalization period experienced chronic wound pain (*p* = 0.03). Tengvall and colleagues [59] reported that the severity of infection was significantly related to pain intensity (*p* < 0.01). Healthcare providers should consider infection or inflammation affects not only pressure ulcer wound but also pain experience.

Age is closely related to incidence of pressure ulcers. Older patients tend to have a higher incidence of pressure ulcers [60,61]. Among the 6.2 million ED admissions per year, elderly patients were at a higher risk of developing pressure ulcers [62]. For example, in one study by Baumgarten and colleagues [63], researchers found that elderly patients admitted to the emergency department had a significantly higher incidence of pressure ulcers (6.2% among 3233 elderly patients who were over age 62). To explain these findings, Jaul and colleagues [64] analyzed factors such as dementia (OR = 3.0, *p* < 0.002), urinary catheter usage (OR = 2.25, *p* < 0.03), low body mass index (OR = 0.92, *p* < 0.02), and anemia (OR = 0.7, *p* < 0.004) to determine if they were associated with pressure ulcer incidence in the elderly. Pressure ulcers are also prevalent in hospitalized children ages 0 to 18 (35% (*N* = 412)), with Stage I being the most common (54%), especially in pediatric patients who use external medical devices (40%) [65]. Kottner and colleagues [66] reported varied prevalence of pressure ulcers (between 2% and 28%) in pediatric patients who use medical devices. According to these reports, elderly and pediatrics who were hospitalized may have high incidence of pressure ulcers and experience higher level of pain related to pressure ulcers. Health care providers should have more consideration about preventive care of pressure ulcers when providing care to elderly and pediatrics.

Age also affects experiences related to pain. In one study, older adults were perceived as being more sensitive to pain than middle-aged adults (*p* < 0.001) and younger adults (*p* = 0.00) [67]. In response to stimuli of pressure pain, the elderly were shown to have lower pain thresholds [68,69]. Therefore, pain perception varies according to age groups. Lillie and colleagues [70] have reported that pain is under assessed or undertreated in older adults. In a study by Ahn *et al.* (2015), age was able to predict pain intensity in a population with pressure ulcers (OR = 0.97, *p* < 0.001) [19]. However, Baharestani and colleagues [71] reported a lack of tools to assess pressure ulcer pain in the neonatal or pediatric population. Collectively, these studies reveal that pain may be inadequately managed in certain age groups. According to the above characteristics of the elderly, their pain level may be expressed lower than their actual pain. Therefore, health care providers should consider age specific characteristics when they manage pain in the pressure ulcer population.

### 3.4. Psychosocial Factors

Pain phenomena are affected by psychological status, stress, sleep, fatigue, catastrophizing, and individual coping styles. Gorecki and colleagues [17] stated that anticipating pain may lead to negative moods, emotions, and anxiety in patients, and that these emotions influence the experiences of pain in patients with pressure ulcers. Anxiety is defined as the “anticipation of impending danger and dread accompanied by restlessness, tension, tachycardia, and breathing difficulty not associated with an apparent stimulus” [45]. In a qualitative study regarding quality of life among patients with pressure ulcers, Gorecki and colleagues [17] described how patients with pressure ulcers experienced emotional distress and anxiety related to their wounds. Similarly, Smeijers and colleagues [72] reported a strong association between anxiety and pain. Indeed, patients with high anxiety levels were more likely to experience pain than controls (OR = 3.09, 95% confidence interval [CI]: 1.52–6.27, *p* = 0.002) even after adjusting for demographic factors among 1279 participants [72].

Depression is “a condition of loss of interest or pleasure in life activities” that leads to “significant impairment in social, work, or other important areas of functioning” [73]. Depression has been studied for several decades as a predictor of pain and a factor that contributes to increased pain. Zakoscielna and Pamrmelee [74] explored predictors of pain variability, and found that more severe baseline depression was related to higher pain variability (β = 0.308, *p* < 0.02). In addition, Ligthart and colleagues [75] reported that depression was strongly associated with more frequent pain and multiple pain sites in 11,787 twin adults.

Several studies found that anger is a strong predictor of pain. Anger refers to “an emotional reaction characterized by extreme displeasure, rage, indignation or hostility” [45]. A literature review by Bruehl and colleagues [76] reported that participants who responded more angrily to pain stimuli had higher levels of chronic pain. In addition, van Middendorp and colleagues [77] reported that people who responded angrily to an event experienced more severe pain (β =−0.09, *p* < 0.001) in a study of 333 women with chronic pain over a period of 28 days.

Stress refers to any stimulus or event that evokes a physiologic stress response [78]. Psychosocial stress plays an important role as a predictor of pain [79,80]. The study of Osteras and colleagues [81] determined a strong association between pain intensity and stress (*r* = 0.40, *p* < 0.01; OR = 1.68, 95% CI: 1.42–1.99) among young people. Hannibal and Bishop [78] reported that exposing chronic physiologic stressors to patients may prolong their pain experiences. Hospitalization causes emotional and physical crisis. Anxiety, stress, and anger are common in hospitalized patients. According to above findings, emotional crisis along with physical crisis may often magnify their pain experience. Emotional crisis influences pain in a similar manner in the hospitalized pressure ulcer population.

Sleep is closely related to pain outcomes. Sleep is defined as “a state marked by reduced consciousness, diminished activity of skeletal muscles, and depressed metabolism” [45]. Sivertsen and colleagues [82] found that insomnia significantly increased the risk of reduced experimental pain tolerance in 10,412 participants. Insomnia often reduced cold pressor pain tolerance (hazard ratio (HR) = 1.52, 1.39, and 1.24 when insomnia occurred once a week, weekly and monthly respectively) [82]. Smith and colleagues [83] discussed that continuous sleep disturbance increased pain and reduced endogenous pain inhibitors among 32 healthy females. Schuh-Hofer and colleagues [84] reported that sleep disturbance increased sensitivity to heat stimuli (*p* < 0.05), blunt pressure stimuli (*p* < 0.05), cold stimuli (*p* < 0.01), and pinprick stimuli (*p* < 0.05) in 14 healthy participants. Schrimpf and colleagues [85] reported that sleep deprivation increased pain perception (*p* = 0.015) among 266 healthy participants through meta-analysis.

Fatigue is “a nonspecific, common subjective feeling of low vitality that disrupts daily functioning” [86]. The relationship between pain and fatigue has been explored in many studies. Pain and fatigue were strongly related (*p* < 0.05) in a population of 250 elderly people with chronic pain [87]. Sturgeon and colleagues [88] described fatigue as a significant factor that increased pain among a chronic pain population of 2487. Van Dartel and colleagues [89] reported that pain and fatigue had a significant positive relationship (β = 2.04, *p* < 0.0001) in 198 patients with chronic pain. Creavin and colleagues [90] investigated the co-occurrence of pain and fatigue, and found that 60% of the 451 participants with chronic pain reported persistent fatigue. In addition, these researchers found that 33% of the 809 participants with persistent fatigue reported chronic pain among 2447 community dwelling Dutch adults. Hospitalized patients commonly experience sleep deprivation and fatigue due to 24 h ongoing care. Sleep and fatigue are closely related to each other. Above findings support that sleep deprivation and fatigue may intensify pain experiences in hospitalized pressure ulcer population.

Catastrophizing is defined as “a negative cognitive and affective process involving magnification of pain-related symptoms, helplessness, pessimism, and rumination about pain” [91]. Quartana and colleagues [92] reported that pain catastrophizing has a significant influence short and long term adverse outcomes of pain. Indeed, it alters the hypothalamic pituitary response, magnifies the activity of the neural region, and decreases endogenous pain inhibition [92]. In addition, using multivariate regression analyses in 59 patients undergoing the treatment of pain, George and Hirsh [93] reported that pain catastrophizing affects pain intensity (β = 0.43, *p* < 0.01) [93].

Coping refers to the cognitive and behavioral adjustments that an individual uses to confront and manage health and daily-life stressors [94]. An individual’s coping strategy is a significant factor in the regulation of pain perception. Moore and colleagues [95] conducted a study to compare pain tolerance between participants who used either acceptance or distraction as a coping method. Their findings suggested that acceptance was associated with better pain tolerance than distraction (*t*(13) = 2.18, *p* < 0.05) [95]. Rejeh and colleagues [96] explored relaxation as an effective technique to decrease pain (*t* = −4.68, *p* = 0.004) in a study of 124 participants over age 65 undergoing surgery. According to above findings, individual pain experiences vary depending on the coping toward their situation. Hospitalization is certainly a crisis for the patients and family. Pressure ulcers are chronic condition and require longer medical treatments. Therefore, coping and catastrophizing may have significant influence in pressure ulcer population and their pain experience.

### 3.5. Environmental Factors

Nurses play an important role in the prevention and treatment of pressure ulcers and any related pain. In one study by Park and colleagues (2014), researchers examined the relationship between staffing and hospital acquired pressure ulcers, and found that increased registered nurse (RN) staffing was associated with a lower rate of pressure ulcers (OR = 0.952–0.950, *p* < 0.011), using the national database of nursing quality indicators from 2008 to 2010 [97]. Their findings were supported by a study by Choi and Staggs [98] that RN-perceived staffing adequacy was significantly associated with unit acquired pressure ulcers (odds = 0.782, 95% CI: 0.647–0.944) in the 57,223 National Database of Nursing Quality Indicators (NDNQI) RN Survey in 2011.

Similarly, Castle and Anderson (2011) reported relationships between staffing variables and quality measures in 2839 nursing homes from 2003 to 2007. Results showed that an increase in RN staffing was related to a decrease in pressure ulcers (−0.46, *p* < 0.001) and improved pain management (−0.53, *p* < 0.05) [99]. These findings indicate that patients’ mobility, activity, and perfusion are facilitated by nurses during hospitalization. In the systematic review of Coleman and colleagues [100] reported that adequate mobility, activity, and perfusion may prevent the development of pressure ulcers. Based on these reports, it is clear that nurses play an important role in the prevention of pressure ulcers and the provision of appropriate pain management in hospitalized patients. Therefore, the nurse/patient ratio should be regarded as one of the environmental factors that affects pain experiences in hospitalized patients with pressure ulcers.

Nurse education and quality of care have been demonstrated to affect the management of pressure ulcers. For example, Aydin and Karadag [101] reported that the educational background of nurses (*p* < 0.004) significantly influenced the prevention and management of sDTI and Stage I pressure ulcers among 243 nurses in Ankara and Turkey. Similarly, Pancorbo-Hidalogo and colleagues [102] reported that knowledge of prevention intervention (*p* < 0.001) and treatment intervention (*p* < 0.001) was greater in RNs with a university degree than in LPNs among 740 nurses in Spain. Based on these reports, it is clear that nurse education level is an environmental factor that can affect the pain experiences of hospitalized patients with pressure ulcers.

During hospitalization, patients may experience pain as a result of pressure ulcer wound treatments and dressings. For example, the negative pressure vacuum assisted system, which is used to treat pressure ulcers, may cause pain [103], as well as wound debridement and cleansing [104]. During treatments such as dressing changes or interventions, patients may experience varying levels of pain [12]. Pieper and colleagues [11] reported that dressing changes cause severe pain and their frequency affects the overall comfort level of patients. Woo and Sibbald [104] also reported that dressing changes aggravate pain in local wounds. Thus, frequency and type of pressure ulcer dressing changes should be included as an environmental factor for hospitalized populations.

In summary, the proposed conceptual framework derived from the biopsychosocial model explains pain in pressure ulcers. Biological, psychological, sociocultural, and environmental factors influence the experience of pain. Possible components in each factor are identified by the literature review for this conceptual framework.

### 3.6. Major Propositions of the Proposed Conceptual Framework

The concepts presented in the proposed model are connected by relational statements. The proposed conceptual framework enables the investigation of concepts using appropriate empirical indicators and provides systematic knowledge of pain phenomena. Several possible hypotheses from the proposed conceptual framework include:

- If other predictors are controlled, pressure ulcer stages/categories will be significantly associated with pain experiences.
- Biological factors, including comorbidities, single nucleotide polymorphisms of specific candidate genes (e.g., OPRM1, COMT), endogenous pain inhibition, specific inflammation markers (e.g., cortisol, beta-endorphin, and cytokines), and age will moderate pain experiences associated with pressure ulcers.
- Sociocultural factors, including ethnicity, discrimination, and social support, will moderate pain experiences associated with pressure ulcers.
- Psychological factors, including anxiety, depression, anger, stress, sleep, fatigue, catastrophizing, and coping strategies, will moderate pain experiences associated with pressure ulcers.
- Environmental factors, including nurse/patients ratio, nurse educational level and frequency/type of dressing changes, will moderate pain experiences associated with pressure ulcers in hospitalized patients.
- These biological, sociocultural, psychological, and environmental factors are assumed to combine in a sequence to influence pain experiences with pressure ulcers.

## 4. Discussion

The proposed conceptual framework, adapted from the biopsychosocial model of pain, uses a multidimensional approach to include possible moderators that influence pain experiences in patients with pressure ulcers. This proposed conceptual framework explains pain phenomena in pressure ulcers in detail. In addition, a future study using this proposed conceptual framework will present empirical evidence that explains the clear association between pain experiences and pressure ulcer stages/categories. The proposed conceptual framework contains factors that affect the pain experiences of hospitalized patients with pressure ulcers. This framework will help health care providers determine the best intervention to prevent, manage, and treat pain related to pressure ulcers. Furthermore, the proposed conceptual framework may be modified to apply to other chronic pain populations as well.

The parent model, which is the “biopsychosocial model of pain”, depicts a bidirectional relationship among the biological, sociocultural, and psychological factors that influence pain. This model uses a multidimensional approach to explain the complexity and variability of pain experiences. It describes pain as a result of complex processes of biopsychosocial interactions. However, it is difficult to define how much these factors may affect pain experiences associated with pressure ulcers. The proposed conceptual framework tries to explain how each factor influences pain experiences associated with pressure ulcers using a linear structure. In addition, environmental factors aimed at hospitalized patients were added to the proposed conceptual framework, which describes a more clear relationship between pain experiences and other concepts related to pressure ulcers in hospitalized patients. The framework also presents possible modifying factors that may contribute to a decrease in pain experiences for this population.

Gorecki and colleagues [17] presented a model that explains how pressure ulcer pain affects a patient’s daily life and how the management of pain is influenced by communication through qualitative and quantitative systematic review. The model presented by Gorecki and colleagues suggested a biopsychosocial approach to pressure ulcer pain and improvements in communication with patients for effective pain management. Our proposed conceptual framework is consistent with their model in terms of how pressure ulcer pain is described from a biopsychosocial perspective. However, the model suggested by Gorecki and colleagues focuses on factors influencing pain management, whereas our conceptual framework focuses on factors that influence the pain experiences of patients in hospital settings, and includes both biopsychosocial and environmental perspectives. Our framework is consistent with the chronic wound associated with pain model suggested by Woo and colleagues [104] because individualized pain is affected by psychological factors, biological factors, and wound treatments. The model proposed by Woo and Sibbald used a variety of chronic wounds to explain pain in general, whereas our proposed conceptual framework focuses on pain associated with pressure ulcers.

Our proposed conceptual framework provides a foundation for future research and practice. First, this conceptual framework directs research by identifying current knowledge regarding the relationship between pressure ulcers and pain, and reducing the number of alternative explanations for findings. In addition, research based on a conceptual model contributes to a more coherent and comprehensive body of knowledge, whereas research without a theory produces isolated information [105,106,107]. Second, future studies that explore the most effective pressure ulcer pain management programs are needed, and these studies should examine pharmacological and non-pharmacological treatment programs (e.g., culturally sensitive, cognitive-behavioral therapy). Third, health care providers who use the proposed conceptual framework will be better able to identify patients who are at risk of experiencing high levels of pain associated with pressure ulcers. Finally, the proposed conceptual framework suggests applicable interventions to decrease pain experiences by manipulating factors such as environmental factors (establishing appropriate nurse/patient ratios and nursing educational levels, and selecting appropriate types of dressings to decrease the frequency of dressing changes), some biological factors (preventing infection and decreasing inflammation), psychological factors (decreasing anxiety and depression, maintaining quiet time to improve sleep quality, and promoting appropriate coping strategies) and sociocultural factors (supporting individual cultural aspects and facilitating family support).

The proposed conceptual framework has limitations. This conceptual framework was built through an extensive literature review and concepts were derived from the biopsychosocial model of pain. Thus, our preliminary conceptual framework aims to provide a comprehensive understanding of the pain experiences of hospitalized patients, and it needs to be tested with hospitalized patients with pressure ulcers. Although it will be difficult to test all factors in this conceptual framework simultaneously, certain factors may be tested in a select sample from the target population.

In terms of pain associated with pressure ulcers, our proposed conceptual framework identifies the important role that healthcare providers play in the prevention, management, and treatment of pressure ulcers and any related pain. Also, the proposed conceptual framework is consistent with current guidelines for the treatment of pressure ulcers, including adequate pain assessment and management [22]. Therefore, healthcare providers should be aware of pain associated with pressure ulcers when they provide care to hospitalized patients with pressure ulcers.

## 5. Conclusions

A comprehensive understanding of pain experiences associated with pressure ulcers is required to increase health related quality of life in patients with pressure ulcers. To date, there is no clear conceptual framework to explain pain experiences associated with pressure ulcers. Based on the review of literature, the proposed conceptual framework was generated from the biopsychosocial model of pain, existing evidence was reviewed based on the biopsychosocial model of pain, and environmental factors were added for the target population. Based on this conceptual framework, several strategies were suggested to decrease the pain experiences of hospitalized patients with pressure ulcers. The proposed conceptual framework will provide an advanced understanding of pain phenomena in hospitalized populations with pressure ulcers. Furthermore, it will guide the development of effective nursing interventions by modifying significant factors that may decrease pain experiences for patients with pressure ulcers and promote their quality of life.

## References

[1] Russo A., Steiner C., Spector W. Hospitalizations Related to Pressure Ulcers among Adults 18 Years and Older, 2006. http://www.hcup-us.ahrq.gov/reports/statbriefs/statbriefs.jsp.

[2] Moore Z. (2013). US medicare data show incidence of hospital-acquired pressure ulcers is 4.5%, and they are associated with longer hospital stay and higher risk of death. Evid. Based Nurs..

[3] Kurtzman E.T., Buerhaus P.I. (2008). New medicare payment rules: Danger or opportunity for nursing?. Am. J. Nurs..

[4] Mattie A.S., Webster B.L. (2008). Centers for medicare and medicaid services’ “never events”: An analysis and recommendations to hospitals. Health Care Manag..

[5] Agency for Healthcare Research and Quality Saving Lives and Saving Money: Hospital-Acquired Conditions Update. http://www.ahrq.gov/professionals/quality-patient-safety/pfp/interimhacrate2014.html.

[6] Park-Lee E., Caffrey C. (2009). Pressure ulcers among nursing home residents: United States, 2004. NCHS Data Brief.

[7] Gorecki C., Brown J.M., Nelson E.A., Briggs M., Schoonhoven L., Dealey C., Defloor T., Nixon J. (2009). Impact of pressure ulcers on quality of life in older patients: A systematic review. J. Am. Geriatr. Soc..

[8] Hopkins A., Dealey C., Bale S., Defloor T., Worboys F. (2006). Patient stories of living with a pressure ulcer. J. Adv. Nurs..

[9] Langemo D.K., Melland H., Hanson D., Olson B., Hunter S. (2000). The lived experience of having a pressure ulcer: A qualitative analysis. Adv. Skin Wound Care.

[10] Gunes U.Y. (2008). A descriptive study of pressure ulcer pain. Ostomy Wound Manag..

[11] Pieper B., Langemo D., Cuddigan J. (2009). Pressure ulcer pain: A systematic literature review and national pressure ulcer advisory panel white paper. Ostomy Wound Manag..

[12] Rastinehad D. (2006). Pressure ulcer pain. J. Wound Ostomy Cont. Nurs..

[13] Spilsbury K., Nelson A., Cullum N., Iglesias C., Nixon J., Mason S. (2007). Pressure ulcers and their treatment and effects on quality of life: Hospital inpatient perspectives. J. Adv. Nurs..

[14] National Pressure Ulcer Advisory Panel Npuap Pressure Ulcer Stages/Categories. http://www.npuap.org/resources/educational-and-clinical-resources/npuap-pressure-ulcer-stagescategories/.

[15] Ratliff C.R., Rodeheaver G.T. (1999). Pressure ulcer assessment and management. Lippincott’s Prim. Care Pract..

[16] Coleman S., Nixon J., Keen J., Wilson L., McGinnis E., Dealey C., Stubbs N., Farrin A., Dowding D., Schols J.M. (2014). A new pressure ulcer conceptual framework. J. Adv. Nurs..

[17] Gorecki C., Closs S.J., Nixon J., Briggs M. (2011). Patient-reported pressure ulcer pain: A mixed-methods systematic review. J. Pain Symptom Manag..

[18] Girouard K., Harrison M.B., van den Kerkof E. (2008). The symptom of pain with pressure ulcers: A review of the literature. Ostomy Wound Manag..

[19] Ahn H., Stechmiller J., Fillingim R., Lyon D., Garvan C. (2015). Bodily pain intensity in nursing home residents with pressure ulcers: Analysis of national minimum data set 3.0. Res. Nurs. Health.

[20] Ahn H., Stechmiller J., Horgas A. (2013). Pressure ulcer-related pain in nursing home residents with cognitive impairment. Adv. Skin Wound Care.

[21] McGinnis E., Briggs M., Collinson M., Wilson L., Dealey C., Brown J., Coleman S., Stubbs N., Stevenson R., Nelson E.A. (2014). Pressure ulcer related pain in community populations: A prevalence survey. BMC Nurs..

[22] National Pressure Ulcer Advisory Panel Prevention and Treatment of Pressure Ulcers: Quick Reference Guide. http://www.npuap.org/wp-content/uploads/2014/08/Updated-10-16-14-Quick-Reference-Guide-DIGITAL-NPUAP-EPUAP-PPPIA-16Oct2014.pdf.

[23] European Pressure Ulcer Advisory Panel Treatment of Pressure Ulcers: Quick Reference Guide. http://www.epuap.org/guidelines/guidelines-old/.

[24] Walker L.O., Avant K.C. (2010). Strategies for Theory Construction in Nursing.

[25] Melzack R., Wall P.D. (1965). Pain mechanisms: A new theory. Science.

[26] Summers S. (2000). Evidence-based practice part 1: Pain definitions, pathophysiologic mechanisms, and theories. J. Perianesthesia Nurs..

[27] Snow A.L., O’Malley K.J., Cody M., Kunik M.E., Ashton C.M., Beck C., Bruera E., Novy D. (2004). A conceptual model of pain assessment for noncommunicative persons with dementia. Gerontologist.

[28] Chung J.W., Wong T.K., Yang J.C. (2000). The lens model: Assessment of cancer pain in a chinese context. Cancer Nurs..

[29] Fillingim R.B. (2005). Individual differences in pain responses. Curr. Rheumatol. Rep..

[30] International Association for the Study of Pain Iasp Taxonomy. http://www.iasp-pain.org/Education/Content.aspx?ItemNumber=1698&amp;navItemNumber=576#Pain.

[31] Bates M.S. (1987). Ethnicity and pain: A biocultural model. Soc. Sci. Med..

[32] Edwards C.L., Fillingim R.B., Keefe F. (2001). Race, ethnicity and pain. Pain.

[33] Lasch K.E. (2000). Culture, pain, and culturally sensitive pain care. Pain Manag. Nurs..

[34] Rahim-Williams F.B., Riley J.L., Herrera D., Campbell C.M., Hastie B.A., Fillingim R.B. (2007). Ethnic identity predicts experimental pain sensitivity in african americans and hispanics. Pain.

[35] Campbell C.M., France C.R., Robinson M.E., Logan H.L., Geffken G.R., Fillingim R.B. (2008). Ethnic differences in diffuse noxious inhibitory controls. J. Pain.

[36] Cruz-Almeida Y., Sibille K.T., Goodin B.R., Petrov M.E., Bartley E.J., Riley J.L., King C.D., Glover T.L., Sotolongo A., Herbert M.S. (2014). Racial and ethnic differences in older adults with knee osteoarthritis. Arthr. Rheumatol..

[37] Williams D.R., Lavizzo-Mourey R., Warren R.C. (1994). The concept of race and health status in America. Public Health Rep..

[38] Garbez R., Puntillo K. (2005). Acute musculoskeletal pain in the emergency department: A review of the literature and implications for the advanced practice nurse. AACN Clin. Issues.

[39] Iyer R.G. (2011). Pain documentation and predictors of analgesic prescribing for elderly patients during emergency department visits. J. Pain Symptom Manag..

[40] Pletcher M.J., Kertesz S.G., Kohn M.A., Gonzales R. (2008). Trends in opioid prescribing by race/ethnicity for patients seeking care in us emergency departments. JAMA.

[41] Bisconti T.L., Bergeman C.S. (1999). Perceived social control as a mediator of the relationships among social support, psychological well-being, and perceived health. Gerontologist.

[42] Lee J.E., Kahana B., Kahana E. (2015). Social support and cognitive functioning as resources for elderly persons with chronic arthritis pain. Aging Ment. Health.

[43] Takai Y., Yamamoto-Mitani N., Abe Y., Suzuki M. (2015). Literature review of pain management for people with chronic pain. Jpn. J. Nurs. Sci..

[44] Ferreira V.M., Sherman A.M. (2007). The relationship of optimism, pain and social support to well-being in older adults with osteoarthritis. Aging Ment. Health.

[45] Anderson D.M., Keith J., Novak P.D., Elliot M.A. (2012). Mosby’s Dictionary of Medicine, Nursing and Health Professions.

[46] Onubogu U.D. (2014). Pain and depression in older adults with arthritis. Orthop. Nurs..

[47] Caporali R., Cimmino M.A., Sarzi-Puttini P., Scarpa R., Parazzini F., Zaninelli A., Ciocci A., Montecucco C. (2005). Comorbid conditions in the amica study patients: Effects on the quality of life and drug prescriptions by general practitioners and specialists. Sem. Arthr. Rheum..

[48] Horjales-Araujo E., Dahl J.B. (2015). Is the experience of thermal pain genetics dependent?. BioMed Res. Int..

[49] Zubieta J.K., Heitzeg M.M., Smith Y.R., Bueller J.A., Xu K., Xu Y., Koeppe R.A., Stohler C.S., Goldman D. (2003). Comt val158met genotype affects mu-opioid neurotransmitter responses to a pain stressor. Science.

[50] Hastie B.A., Riley J.L., Kaplan L., Herrera D.G., Campbell C.M., Virtusio K., Mogil J.S., Wallace M.R., Fillingim R.B. (2012). Ethnicity interacts with the oprm1 gene in experimental pain sensitivity. Pain.

[51] Fillingim R.B., Kaplan L., Staud R., Ness T.J., Glover T.L., Campbell C.M., Mogil J.S., Wallace M.R. (2005). The A118G single nucleotide polymorphism of the mu-opioid receptor gene (OPRM1) is associated with pressure pain sensitivity in humans. J. Pain.

[52] Diatchenko L., Slade G.D., Nackley A.G., Bhalang K., Sigurdsson A., Belfer I., Goldman D., Xu K., Shabalina S.A., Shagin D. (2005). Genetic basis for individual variations in pain perception and the development of a chronic pain condition. Hum. Mol. Genet..

[53] Bruehl S., Burns J.W., Gupta R., Buvanendran A., Chont M., Schuster E., France C.R. (2014). Endogenous opioid inhibition of chronic low-back pain influences degree of back pain relief after morphine administration. Reg. Anesthesia Pain Med..

[54] Gatchel R.J., Peng Y.B., Peters M.L., Fuchs P.N., Turk D.C. (2007). The biopsychosocial approach to chronic pain: Scientific advances and future directions. Psychol. Bull..

[55] Starkweather A.R., Lyon D.E., Schubert C.M. (2013). Pain and inflammation in women with early-stage breast cancer prior to induction of chemotherapy. Biol. Res. Nurs..

[56] DeVon H.A., Piano M.R., Rosenfeld A.G., Hoppensteadt D.A. (2014). The association of pain with protein inflammatory biomarkers: A review of the literature. Nurs. Res..

[57] White R.J. (2009). Wound infection-associated pain. J. Wound Care.

[58] Juozapaviciene L., Rimdlka R., Karbonskiene A. (2012). Problem with the post burn wound pain: Chronic profiles. EWMA J..

[59] Tengvall O.M., Bjornhagen V.C., Lindholm C., Jonsson C.E., Wengstrom Y. (2006). Differences in pain patterns for infected and noninfected patients with burn injuries. Pain Manag. Nurs..

[60] Vangilder C., Macfarlane G.D., Meyer S. (2008). Results of nine international pressure ulcer prevalence surveys: 1989 to 2005. Ostomy Wound Manag..

[61] National Pressure Ulcer Advisory Panel (2012). Pressure Ulcers: Prevalence, Incidence and Implications for Future.

[62] Niska R., Bhuiya F., Xu J. (2010). National hospital ambulatory medical care survey: 2007 emergency department summary. Natl. Health Stat. Rep..

[63] Baumgarten M., Margolis D.J., Localio A.R., Kagan S.H., Lowe R.A., Kinosian B., Holmes J.H., Abbuhl S.B., Kavesh W., Ruffin A. (2006). Pressure ulcers among elderly patients early in the hospital stay. J. Gerontol. Ser. A Biol. Sci. Med. Sci..

[64] Jaul E., Calderon-Margalit R. (2015). Systemic factors and mortality in elderly patients with pressure ulcers. Int. Wound J..

[65] Schlüer A.B., Halfens R.J., Schols J.G.A. (2012). Pediatric pressure ulcer prevalence: A multicenter, cross-sectional, point prevalence study in switzerland. Ostomy Wound Manag..

[66] Kottner J., Wilborn D., Dassen T. (2010). Frequency of pressure ulcers in the paediatric population: A literature review and new empirical data. Int. J. Nurs. Stud..

[67] Wandner L.D., Scipio C.D., Hirsh A.T., Torres C.A., Robinson M.E. (2012). The perception of pain in others: How gender, race, and age influence pain expectations. J. Pain.

[68] Cole L.J., Farrell M.J., Gibson S.J., Egan G.F. (2010). Age-related differences in pain sensitivity and regional brain activity evoked by noxious pressure. Neurobiol. Aging.

[69] Lautenbacher S., Kunz M., Strate P., Nielsen J., Arendt-Nielsen L. (2005). Age effects on pain thresholds, temporal summation and spatial summation of heat and pressure pain. Pain.

[70] Lillie A.K., Read S., Mallen C., Croft P., McBeth J. (2013). Musculoskeletal pain in older adults at the end-of-life: A systematic search and critical review of the literature with priorities for future research. BMC Palliat. Care.

[71] Baharestani M.M., Ratliff C.R. (2007). Pressure ulcers in neonates and children: An npuap white paper. Adv. Skin Wound Care.

[72] Smeijers L., van de Pas H., Nyklicek I., Notten P.J., Pedersen S.S., Kop W.J. (2014). The independent association of anxiety with non-cardiac chest pain. Psychol. Health.

[73] Papadakis M., McPhee S.J., Rabow M.W. (2015). Current Medical Diagnosis and Treatment.

[74] Zakoscielna K.M., Parmelee P.A. (2013). Pain variability and its predictors in older adults: Depression, cognition, functional status, health, and pain. J. Aging Health.

[75] Ligthart L., Visscher C.M., van Houtem C.M., Geels L.M., Vink J.M., de Jongh A., Boomsma D.I. (2014). Comorbidity among multiple pain symptoms and anxious depression in a dutch population sample. J. Pain.

[76] Bruehl S., Chung O.Y., Burns J.W. (2006). Anger expression and pain: An overview of findings and possible mechanisms. J. Behav. Med..

[77] Van Middendorp H., Lumley M.A., Moerbeek M., Jacobs J.W., Bijlsma J.W., Geenen R. (2010). Effects of anger and anger regulation styles on pain in daily life of women with fibromyalgia: A diary study. Eur. J. Pain.

[78] Hannibal K.E., Bishop M.D. (2014). Chronic stress, cortisol dysfunction, and pain: A psychoneuroendocrine rationale for stress management in pain rehabilitation. Phys. Ther..

[79] Shapiro M.A., Nguyen M.L. (2010). Psychosocial stress and abdominal pain in adolescents. Ment. Health Fam. Med..

[80] White K.S., Farrell A.D. (2006). Anxiety and psychosocial stress as predictors of headache and abdominal pain in urban early adolescents. J. Pediatr. Psychol..

[81] Osteras B., Sigmundsson H., Haga M. (2015). Perceived stress and musculoskeletal pain are prevalent and significantly associated in adolescents: An epidemiological cross-sectional study. BMC Public Health.

[82] Sivertsen B., Lallukka T., Petrie K.J., Steingrimsdottir O.A., Stubhaug A., Nielsen C.S. (2015). Sleep and pain sensitivity in adults. Pain.

[83] Smith M.T., Edwards R.R., McCann U.D., Haythornthwaite J.A. (2007). The effects of sleep deprivation on pain inhibition and spontaneous pain in women. Sleep.

[84] Schuh-Hofer S., Wodarski R., Pfau D.B., Caspani O., Magerl W., Kennedy J.D., Treede R.D. (2013). One night of total sleep deprivation promotes a state of generalized hyperalgesia: A surrogate pain model to study the relationship of insomnia and pain. Pain.

[85] Schrimpf M., Liegl G., Boeckle M., Leitner A., Geisler P., Pieh C. (2015). The effect of sleep deprivation on pain perception in healthy subjects: A meta-analysis. Sleep Med..

[86] Addington A.M., Gallo J.J., Ford D.E., Eaton W.W. (2001). Epidemiology of unexplained fatigue and major depression in the community: The baltimore eca follow-up, 1981–1994. Psychol. Med..

[87] Erturk M., Yildirim Y., Kilic S.P., Ozer S., Aykar F.S. (2015). Pain and fatigue in elderly cancer patients: Turkey example. Holist. Nurs. Pract..

[88] Sturgeon J.A., Darnall B.D., Kao M.C., Mackey S.C. (2015). Physical and psychological correlates of fatigue and physical function: A collaborative health outcomes information registry (choir) study. J. Pain.

[89] Van Dartel S.A., Repping-Wuts J.W., van Hoogmoed D., Bleijenberg G., van Riel P.L., Fransen J. (2013). Association between fatigue and pain in rheumatoid arthritis: Does pain precede fatigue or does fatigue precede pain?. Arthr. Care Res..

[90] Creavin S.T., Dunn K.M., Mallen C.D., Nijrolder I., van der Windt D.A. (2010). Co-occurrence and associations of pain and fatigue in a community sample of dutch adults. Eur. J. Pain.

[91] Buenaver L.F., Edwards R.R., Smith M.T., Gramling S.E., Haythornthwaite J.A. (2008). Catastrophizing and pain-coping in young adults: Associations with depressive symptoms and headache pain. J. Pain.

[92] Quartana P.J., Campbell C.M., Edwards R.R. (2009). Pain catastrophizing: A critical review. Expert Rev. Neurother..

[93] George S.Z., Hirsh A.T. (2009). Psychologic influence on experimental pain sensitivity and clinical pain intensity for patients with shoulder pain. J. Pain.

[94] Pelaez-Ballestas I., Boonen A., Vazquez-Mellado J., Reyes-Lagunes I., Hernandez-Garduno A., Goycochea M.V., Bernard-Medina A.G., Rodriguez-Amado J., Casasola-Vargas J., Garza-Elizondo M.A. (2015). Coping strategies for health and daily-life stressors in patients with rheumatoid arthritis, ankylosing spondylitis, and gout: Strobe-compliant article. Medicine.

[95] Moore H., Stewart I., Barnes-Holmes D., Barnes-Holmes Y., McGuire B.E. (2015). Comparison of acceptance and distraction strategies in coping with experimentally induced pain. J. Pain Res..

[96] Rejeh N., Heravi-Karimooi M., Vaismoradi M., Jasper M. (2013). Effect of systematic relaxation techniques on anxiety and pain in older patients undergoing abdominal surgery. Int. J. Nurs. Pract..

[97] Park S.H., Boyle D.K., Bergquist-Beringer S., Staggs V.S., Dunton N.E. (2014). Concurrent and lagged effects of registered nurse turnover and staffing on unit-acquired pressure ulcers. Health Serv. Res..

[98] Choi J., Staggs V.S. (2014). Comparability of nurse staffing measures in examining the relationship between rn staffing and unit-acquired pressure ulcers: A unit-level descriptive, correlational study. Int. J. Nurs. Stud..

[99] Castle N.G., Anderson R.A. (2011). Caregiver staffing in nursing homes and their influence on quality of care: Using dynamic panel estimation methods. Med. Care.

[100] Coleman S., Gorecki C., Nelson E.A., Closs S.J., Defloor T., Halfens R., Farrin A., Brown J., Schoonhoven L., Nixon J. (2013). Patient risk factors for pressure ulcer development: Systematic review. Int. J. Nurs. Stud..

[101] Aydin A.K., Karadag A. (2010). Assessment of nurses’ knowledge and practice in prevention and management of deep tissue injury and stage i pressure ulcer. J. Wound Ostomy Cont. Nurs..

[102] Pancorbo-Hidalgo P.L., Garcia-Fernandez F.P., Lopez-Medina I.M., Lopez-Ortega J. (2007). Pressure ulcer care in spain: Nurses’ knowledge and clinical practice. J. Adv. Nurs..

[103] Chiummariello S., Guarro G., Pica A., Alfano C. (2012). Evaluation of negative pressure vacuum-assisted system in acute and chronic wounds closure: Our experience. G. Chir..

[104] Woo K.Y., Sibbald R.G. (2008). Chronic wound pain: A conceptual model. Adv. Skin Wound Care.

[105] Fawcett J., DeSanto-Madeya S. (2012). Contemporary Nursing Knowledge: Analysis and Evaluation of Nursing Models and Theories.

[106] Meleis A.I. (2011). Theoretical Nursing: Development and Progress.

[107] Burns N.B., Grove S.K. (2005). The Practice of Nursing Research: Conduct, Critique, and Utilization.

